# Genetic constraints on protein loop evolution: out-of-frame stop codons limit *Bacillus subtilis* stationary-phase mutagenesis

**DOI:** 10.3389/fmolb.2026.1714028

**Published:** 2026-03-09

**Authors:** Carmen Vallin, Mario Pedraza-Reyes, Eduardo Robleto

**Affiliations:** 1 Biochemistry and Molecular Pharmacology Department, NYU Grossman School of Medicine or NYU Langone, New York, NY, United States; 2 Division of Natural and Exact Sciences, Department of Biology, University of Guanajuato, Guanajuato, Mexico; 3 School of Life Sciences, University of Nevada, Las Vegas, NV, United States

**Keywords:** frameshift mutation, microbiology, mutagenesis, protein evolution, transcription-coupling repair factor

## Abstract

Insertion and deletion (indel) events in protein-coding DNA are associated with loss-of-function frameshift mutations. *In silico* studies have found that protein-coding regions are littered with out-of-frame stop codons (OSCs) which may restrict OSC frameshifts from becoming non-functional polypeptides by prematurely terminating translation. However, the effect of OSCs on protein variation has not been tested *in vivo*—specifically when OSCs are found in coding regions characterized by relaxed selection like protein loops. Here, we examined the formation of indel mutations in DNA encoding the *Bacillus subtilis* loop region of the ornithine carbamoyltransferase (Otc) protein by constructing a gain-of-function genetic system in strains with or without OSCs (+OSC and −OSC, respectively). The use of a gain-of-function selection system and a DNA region coding for a protein loop in which a wide spectrum of amino acids can restore function allowed us to maximize the spectrum of indels we could detect. We found that the +OSC strain had a lower indel frequency than the −OSC strain. Furthermore, indel events in the −OSC strain were not possible in the +OSC strain because they would shift the OSC into frame and terminate translation. By analyzing all indel events in both background strains, we found that the −OSC strain had a broader indel spectrum and led to more amino acid variation in the protein loop of Otc than did the +OSC strain. This variation was not due to GC content or the presence of homopolymers. Indel formation was dependent on the transcription-coupled repair factor Mfd and increased with elevated transcription. In addition to promoting the premature translation termination of non-functional peptides, this study provides the first *in vivo* evidence that OSCs are DNA features that restrict genetic changes and constrict the evolution of new protein domains like those that evolve from protein loops.

## Introduction

Maintaining the correct open reading frame (ORF) sequence is essential for functional protein production, which is essential for life. However, changes to the ORF that lead to new protein folds and domains are also critical to the evolutionary process. Frameshifts are DNA mutations that disrupt the ORF by the insertion or deletion (indels) of nucleotides. Like single nucleotide changes (base substitutions), frameshifts produce genetic variations that, while important for evolution, are also more likely to have deleterious effects because disrupting ORFs often produces truncated, non-functional products or toxic peptides. Thus, frameshifts have varying costs to the cell.

In studies of symbiotic bacterial genomes, indels were more prevalent in pseudogenes and intergenic regions than in protein-coding DNA, suggesting that indels may be tolerated in genome hotspots (Moran et al., 2009). Indels were also observed at the end of genes, minimizing ORF disruption, and in DNA regions that encode protein loops. Protein loops are amino acid sequences that connect secondary structural elements such as alpha-helices and beta-sheets. Importantly, protein loops and their encoding DNA are thought to be indel hotspots and substrates for the evolution of new protein folds and domains that confer selective advantages. This assertion is supported by large variations in the length and amino acid composition of protein loops observed within conserved enzymes (Blouinet al., 2004). If protein loops are indeed hotspots for indel events, what factors influence their evolution?

Coding sequences may contain out-of-frame stop codons (OSC) scattered throughout the ORF. OSCs occur when consecutive amino acid codons form one of three possible translational stop codons in a reading frame different from the coding sequence, creating premature or hidden stop codons, such as CTG AAA. OSCs are thought to protect against indels that cause energetically costly translational frameshifts and produce long, misfolded, or even cytotoxic peptides (Seligmann and Pollock, 2004). Therefore, in the context of indels that disrupt the ORF, we asked whether there is a cost or benefit associated with the presence of OSCs. Furthermore, what are their implications in the evolution of protein domains under relaxed selection? *In silico* experiments in bacteria suggest that the presence of OSCs limits gene recombination events from occurring in protein-coding genes and the formation of variants that broaden metabolism (Wong et al., 2008). These experiments reported that metabolically diverse bacteria and some pathogens have fewer OSCs in their genomes independent of GC content (Wong et al., 2008). These studies suggest that the presence of OSCs in the genome constrains protein variability and ultimately can slow protein evolution and adaptation.

Most studies examining the effects of OSCs on protein evolution followed *in silico* approaches. However, we used an experimental gain-of-function selection-based system to determine whether OSCs influence the formation of spontaneous indel events. This assay employed a defective *argF* gene; we introduced a +1 frameshift mutation into the DNA region encoding the protein loop ArgF^37–47^. The *argF* gene encodes ornithine carbamoyltransferase (Otc), a protein essential for arginine biosynthesis in *Bacillus subtilis.* We next designed two constructs (+OSC and −OSC) and tested their effect on the *B. subtilis* stationary-phase cell’s ability to accumulate gain-of-function indels that would restore Otc function and arginine prototrophy (Arg^−^ → Arg^+^ on minima media plates). Our results showed that the −OSC has a higher indel frequency, indicated by more Arg^+^ colonies. After sequence analysis of all Arg^+^ colonies, we found indel events that were frame-permissive in both strains. The −OSC strain accumulated more diverse mutations than the +OSC construct, which expanded and contracted the Otc loop region in stationary-phase cells. Furthermore, consistent with our previous research which identified factors that contribute to stationary-phase mutagenesis, the formation of Arg^+^ colonies depended on Mfd and was promoted by transcription (Ross et al., 2006; Martin et al., 2011). Mfd is characterized as a protein that mediates the repair of lesions that stall the RNAP during transcription of the template strand by directly dislocating the RNAP, which uncovers the lesion and recruits the nucleotide excision repair system; this pathway is referred to as “transcription-coupled repair” (TCR) (Selby et al., 2023). However, our research and that of others has shown that Mfd functions beyond traditional TCR and can interact with other repair pathways to promote mutations, including indels (Gómez-Marroquín et al., 2016; Villegas-Negrete et al., 2017), and has undefined roles in gene expression (Ho et al., 2018; Martin et al., 2021; Ragheb et al., 2021). This study provides the first *in vivo* experimental evidence that OSCs are DNA features that promote the termination of gene products that have an altered reading frame. However, OSCs also constrain mutational events which lead to protein variability and evolution.

## Materials and methods

### Cloning and construction of OSC strains

The +OSC and −OSC strains were constructed for this study (see Table 1) and were made using PCR mutagenesis in which the primers contained the changes of interest (see Table 2 for the primers). The +OSC strain was constructed to contain the +1T frameshift downstream of codon CAA-Q37 while keeping the two OSCs naturally found in the *B. subtilis argF* sequence intact. The −OSC strain was constructed to contain the same +1 frameshift downstream of codon Q37, and additional synonymous changes were made to codons to remove the OSCs but retain the amino acid sequence. In brief, we designed primers with the desired changes to *argF* that would introduce the +1 frameshift and remove the OSCs (Table 2). To construct the +OSC strain, primers (1) and (6) were first used to amplify a 942-bp segment using NEB Phusion High-Fidelity DNA Polymerase with *B. subtilis* genomic DNA as the template. After purifying the PCR product using a Promega PCR clean-up kit, the 942-bp fragment was used as the template for PCR 2 using Phusion High-Fidelity DNA Polymerase. PCR 2 used primers (2) and (6) to amplify a 965-bp fragment. We then used primers (3) and (4) to amplify a 593-bp fragment with Phusion High-Fidelity DNA Polymerase. All PCR reactions were cleaned up as described above, and the concentration was determined. A 1:1 ratio of fragments 593-bp and 965-bp were combined in a new PCR reaction, mixed with NEB GoTaq reaction reagents, and amplified in seven cycles of denaturing, annealing, and extension. During these steps, two products would denature into single-strand DNA, anneal over 21b (overlap), and amplify during extension. After seven cycles, primers (5) WT argF F (SalI site) and (6) WT argF R (SphI site) were added to the reaction, and PCR proceeded for 16 cycles. The 1,056-bp product was resolved on a gel, excised, and purified. The product was cloned into pdr111 amyE-hyper-SPANK (containing a spectinomycin resistance cassette) plasmid using the SalI and SphI restriction sites and transformed into CV4000 *(arg::neo)* and YB9801 *(mfd::tc)* using standard *B. subtilis* transformation techniques. Transformants were selected on tryptose blood agar base medium (TBAB; Difco Laboratories, Detroit, Mich.) plates containing spectinomycin (YB955) and tetracycline (YB9801) antibiotics. The strains were verified by Sanger sequencing. The same strategy was used to construct the −OSC strain, with primers 1, 2, and 4 substituted with 7, 8, and 9 respectively.

**TABLE 1:** Strains and plasmids.

Strain	Relevant genotype	Reference source
CV101 (+OSC)	*metB5, hisC952, leuC427, arg::neo* *amyE::hyper-SPANK-argF + OSC*	This study
CV102 (+OSC mfd)	*metB5, hisC952, leuC427, arg::neo, mfd::tc* *amyE::hyper-SPANK-argF + OSC*	This study
CV103 (−OSC)	*metB5, hisC952, leuC427, arg::neo* *amyE::hyper-SPANK-argF-OSC*	This study
CV104 (−OSC mfd)	*metB5, hisC952, leuC427, arg::neo, mfd::tc* *amyE::hyper-SPANK-argF-OSC*	This study
CV4000	*metB5, hisC952, leuC427, arg::neo*	Martin et al. (2019)
YB955	*metB5, hisC952, leuC427*	Sung and Yasbin (2002)
YB9801	*metB5, hisC952, leuC427, mfd::tc*	Ross et al. (2006)
pDR111 plasmid	*amyE-hyper-SPANK (spec)*	Rudner Lab

**TABLE 2:** Primers used to construct +/−OSC strains.

#	ID	Sequence
1	+OSC insert 1 F	GGTGAGCTGAAACAATAACAAAATTCAGCCTATATTCCATGGC
2	+OSC insert 2 F	TAAACGCCTTGCTCGCAGAAGCCGGTGAGCTGAAACAATAACAAA
3	argF ups F	TGCTGGCGGGAGCGTCCTTAAAAGATCTAAATCC
4	argF + OSC ovhg R	CTTCTGCGAGCAAGGCGTTTATATCTTCTTCACTAAGGTCC
5	WT argF R (SphI site)	ACCA GCATGC CCTCCTTTTCCTTTTTGCTGTAGTATGC
6	WT argF F (SalI site)	GATGTCGACTAAAAAGGAAGTGGCATCATGCACACAGTGACGCAA
7	−OSC insert1 F	GCCGGAGAACTGAAACAATAACAAAATTCAACCTATATTCCATGCC
8	−OSC insert2 F	AACGCCTTGCTAGCAGAAGCCGGAGAACTCAAACAATAACAAAATACAACCT
9	argF −OSC ovhg R	CTTCTGCTAGCAAGGCGTTTATATCTTCTTCACTAAGGTCC

**TABLE 3:** Sequencing primers.

ID	Sequence
Pspac F	ACTTTATCTACAAGGTGTGGCATAATGTGTGGAATT
WT argF rev (SphI site)	ACCA GCATGC CCTCCTTTTCCTTTTTGCTGTAGTATGC
argF sequencing 1 RV	TTGCCGTATCAGCGACAGTCTCACCGC
argF sequencing 2 RV	TCCGCCTGCTGGAACACTGCAGAGTT
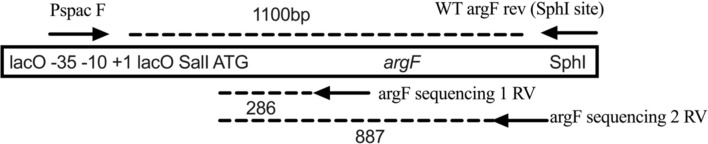	

### Stationary-phase mutagenesis assay

The protocol established by Sung and Yasbin (2002) was followed. Strains were streaked onto TBAB plates containing neomycin and spectinomycin for the +OSC and −OSC strains and tetracycline when working with the Mfd counterpart; they were incubated overnight at 37 °C. The next day, a colony from the TBAB plate was used to inoculate 2 mL of Penassay Broth (PAB; antibiotic medium 3, Difco Laboratories, Sparks, MD, USA), which was grown overnight at 37 °C with aeration. An aliquot of the overnight culture was then added to a 250 mL Erlenmeyer culture flask containing 10 mL of PAB medium and 10 μL of 1,000× Ho-Le trace elements (Sung and Yasbin, 2002). Cells were grown at 37 °C with aeration (250 rpm) to stationary phase. Growth was monitored with a spectrophotometer measuring O.D. at 600 nm (OD600). Cells were harvested 90 min after the end of exponential growth and centrifuged at 4,000 rpm for 5 min; after decanting the supernatant, the cells were resuspended in Spizizen minimal salts (SMS). This process was repeated twice. Initial cell titers were determined by serial dilution, and 100 μL of cells were plated on minimal media supplemented with 100 μg/mL arginine. Colonies were counted after 48 h of incubation at 37 °C. After that, 100 μL aliquots were plated on minimal media containing 0.1 mM IPTG and no arginine (n = 5) and incubated for 12–14 days at 37 °C. Plates were scored daily for the appearance of Arg^+^ colonies, and initial titers were used to normalize the cumulative number of revertants per day to the number of the CFU plated. To assay background cell survival, viability was tracked by taking agar plugs every other day, serial diluting, and plating on minima media supplemented with 100 g/mL arginine. The experiments were repeated four times.

### Fluctuation test

We performed a fluctuation test on each strain to determine the mutation rates for arginine prototrophy during growth. In brief, an aerated culture grown overnight in 2 mL of PAB medium at 37 °C, was diluted 1:10 in 1× SMS, vortexed, and diluted 1:10 again in this solution. Aliquots of the second 1× SMS solution (0.5 mL) were added to 49.5 mL of PAB supplemented with 0.1 mM IPTG. Cells were vortexed, and 1 mL of the 10^–4^ diluted cell mixture was dispensed into 40 different 18 mm test tubes. Cells were grown for 12 h at 37 °C with aeration (250 rpm). Each test tube culture was pelleted, washed, resuspended in 100 µL of 1× SMS, and individually plated onto minimal media lacking arginine with 0.1 mM IPTG. Separately, 1 mL aliquots of the 10^–4^ culture, individually dispensed into individual tubes, were serially diluted and plated on minimal media supplemented with 100 μg/mL arginine to determine cell count. After incubating at 37 °C for 48 h, the colonies were counted. Mutation rates were determined using the Lea–Coulson method of the median (Foster, 2006; Rosche and Foster, 2000).

### Arg^+^ sequencing

Arg^+^ colonies that appeared between days 5 and 12 in the SPM were picked and patched on minimal media lacking arginine and containing 0.1 mM IPTG to ensure stable mutations. Arg^+^ colonies were taken from four separate SPM experiments. After patches were grown for 48 h s, a small sample was then grown in minimal broth; genomic DNA was extracted and used for Gotaq PCR using Pspac F primer and WT argF rev (SphI site). PCR products were sent to Functional Biosciences (Madison, WI) for Sanger sequencing. Routinely, argF sequencing 1RV was chosen as the sequencing primer as it covered 286 bp of the gene and the promoter. To ensure capture of the whole gene argF sequencing, 2RV was also sequenced for some Arg^+^ mutants. See Table 3 for list of sequencing primers.

### Sequence reconstruction analysis

We took an indel event that occurred in the +OSC and −OSC strain and reconstructed it in the opposite strain by inserting the sequence at the same site at which it occurred in the parent strain. We then translated the sequence to determine its effect on the protein loop composition.

### Statistical analysis

Significance was determined using an unpaired Student’s t-test to compare two strains and an ordinary one-way analysis of variance to compare more than two strains. Each comparison had a minimum of three trials.

### Protein alignment analysis

The *B. subtilis* 319 amino acid ArgF protein sequence was input into pBLAST as the query sequence and blasted into the UniProtKB/Swiss-Prot (SwissProt) database. The output identified 100 ArgF amino acids (complete list in supplemental). We used the pBLAST distance tree results to identify the domain and phylum that each sequence fell into. We then exported the pBLAST multiple sequence alignment into SnapGene for further analysis. Additionally, positions 57–89 from the alignment corresponding to the protein loop region were then imported into WebLogo (Crooks et al., 2004) to measure amino acid conservation at those positions (bits).

### Generating protein loop structures

We took four entries out of the 100 that were representative of different loop lengths and phylogenetic groups and looked at their Swiss-Model structures under the 2D structure mode. The 2D structure identified and colored alpha-helices (blue/purple) and beta-sheets (green). We then located the loop sequence we had identified for each entry using the protein alignment and confirmed that it was indeed found in a loop in the model. We zoomed in on the loop and labeled the first and last position. The Swiss-Model Identifiers for the four entries were P18186 (OTC_BACSU), Q5N1N8 (OTC_SYNP6), Q8TKT5 (OTC_METAC), and Q2G3J8 (OTC_NOVAD).

### Calculating the percentage likelihood of forming OSCs in a DNA-based on amino acid sequence

The percentage likelihood of forming OSCs was calculated in two ways. First, we considered all possible codon pairs at positions 35, 36, 64, and 65 (referencing the Weblogo figure, which shows the OSCs of interest) and the likelihood of forming an OSC, then we considered the frequency of each amino at each given position. This analysis considered all possible amino acid pairs for positions 35, 36, 64, and 65. In the second method, we calculated the likelihood of an OSC for each of the 100 sequences based on their specific amino acid pairs at these positions and then found the likelihood for these 100. This analysis excluded non-occurring amino acid pairs.

## Results

### OSCs flank flexible protein loops across different phyla

**FIGURE 1:**
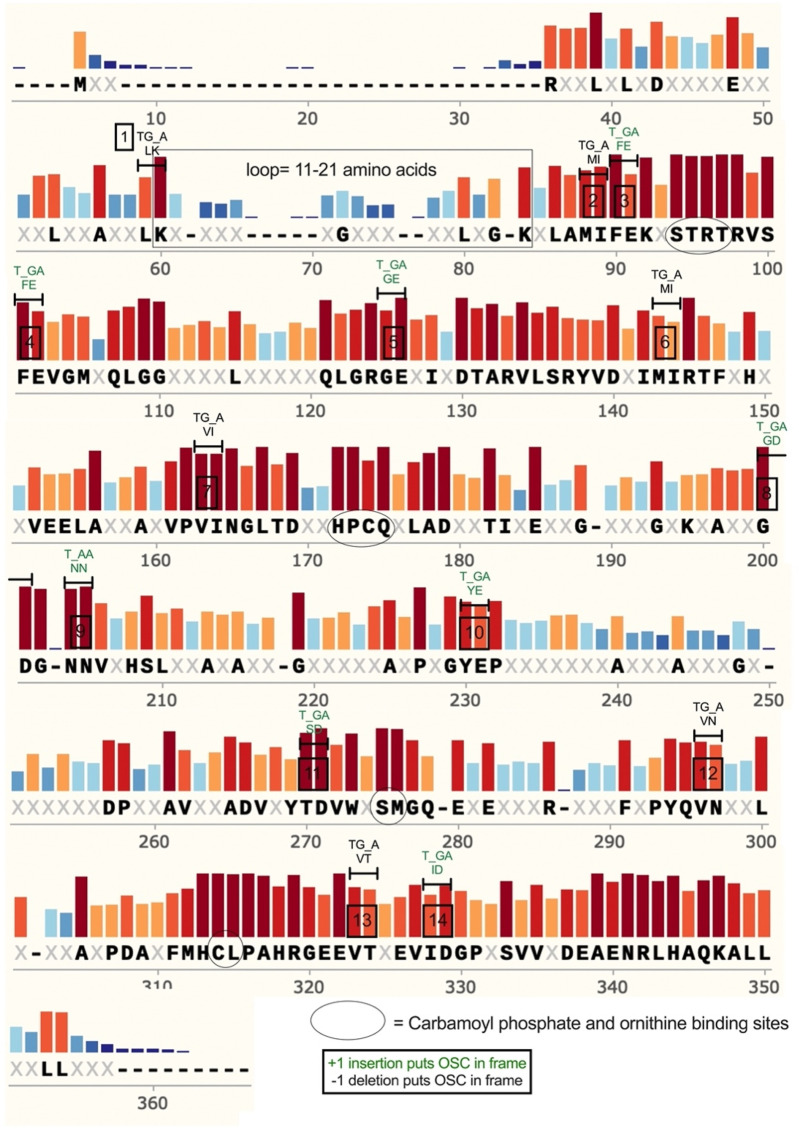
Summarized results from a protein sequence alignment of 100 Otc proteins of diverse origin, including *Bacillus subtilis*. Bar height and color (red) represent how conserved the amino acid was in the 100 sequences. If more than 50% of the sequences had the same amino acid, there is a bold letter underneath the bar. Some 14 OSCs found in *B. subtilis* are overlayed over the alignment labeled “1–14” in a black box over the bars. Black circles show important substrate binding sites. The Otc protein loop region is boxed in and spans K_60_–K_84_ in the alignment.

In *Bacillus subtilis* and other bacteria, the *argF* gene encodes ornithine carbamoyltransferase (Otc), a protein that catalyzes the sixth step in arginine biosynthesis. We previously showed that engineered mutations in *argF* can render bacterial cells auxotrophic for arginine and also score mutations that restore growth (Ermi et al., 2021; Martin et al., 2019). After identifying 46 OSCs in *argF* (Supplementary Figures S1 and S2), we determined whether OSCs occur in conserved protein regions by analyzing *B*. *subtilis* OSCs and 100 Otc protein sequences from diverse prokaryotes (list found S10). We found 14/46 (∼30%) *of B. subtilis* OSCs mapped to conserved amino acid codons (Figure 1), with 13 of the 14 conserved OSCs being opal stops (TGA) and one an ochre stop (TAA) (Supplementary Figure S3). An amino acid was defined as conserved if >50% of protein sequences had the same amino acid.

**FIGURE 2:**
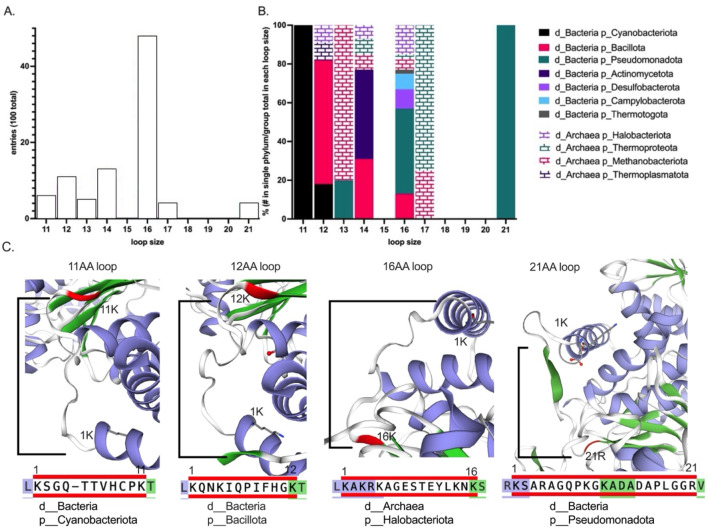
Taxonomic analysis of Otc loop length. The loop length was determined for 100 sequences, starting with the amino acid residue K at position 36 and ending with K at position 47 in *Bacillus subtilis* Otc, corresponding to positions K60–K84 in the alignment. The Genome Taxonomy Database was used to identify the phylum for each entry. **(A)** Contribution of each group to the total count at different loop lengths. **(B)** The percentage for each loop length was calculated by dividing the contribution of each group by the total within that group. “d” indicates domain, while “p” indicates the phylum to which the sequences belong. Bacteria are represented by solid bars, while Archaea are represented by striped bars. **(C)** Four representatives of the 100 sequences, showcasing different loop sizes from various phylogenetic groups. Structures generated using Swiss-Model and are zoomed to show the OTC loop. The blue/purple colors in the figure represent alpha-helices, while green signifies beta-sheets. The first and last positions of the loop are labeled, with the last indicated in red. Below the OTC loop diagram, the loop sequence is presented, similarly colored to illustrate the transition from an alpha helix to a beta-sheet. Diagrams are labeled with their domain and phylum.

Using publicly available Otc structures, we then asked if OSC sites in *B. subtilis* occur in particular protein features such as near substrate binding sites or protein loop regions (see supplemental Otc structure analysis). We found that several OSCs flanked an Otc protein region that varied in sequence and length corresponding to a loop region spanning amino acids K36–K47 in the *B. subtilis* protein and regions 60–84 in the alignment (Figure 1, see boxed region). Analysis of 100 sequences that included bacteria and Archaea indicated that loop length varied from 11 to 21 amino acids and variation tracked with taxonomic groups (Figures 2A and B). *Bacillus* species belonging to phylum Bacillota (pink bar) trended toward decreased loop size (<16 amino acids), similar to cyanobacteria, with a loop length of 11 amino acids (black bar). In comparison, Pseudomonadota (green) had loop sizes of 21 amino acids, although these loops were less frequent (Figures 2A and B). Of these 100 sequences, most had loop lengths that were 16 amino acids long (Figure 2A). While we assumed that we identified a loop region for the 100 sequences based on available Otc structures and because this region fitted the characteristics of a loop region (flexible in sequence and length), we followed this analysis up by looking at the Swiss-Model structures of a few representatives. We were able to verify that the alignment was a good predictor of protein loops (Figure 2C). Interestingly, a small beta-sheet consisting of three amino acids was predicted to be in the “loop” region of the Pseudomonadota representative (Figure 2C, last panel). This observation supports the idea that loops can evolve and potentially form new 2D structures and domains that are important for protein evolution (Blouin et al., 2004).

Since protein loops tolerate indel events and are postulated to be hotspots for protein evolution (Blouin et al., 2004), we asked whether *Bacillus* OSCs flanking the loop region are conserved among prokaryotes, suggesting that they have a biological function. We analyzed the frequency of each amino acid in 100 prokaryotic Otcs to determine the codon options in positions where OSCs were present. The results suggest that OSCs immediately flanking the loop region are conserved and negatively correlated to loop size (Supplementary Figures S5,S6). We became interested in the OSC preceding the *B. subtilis* Otc loop corresponding to L_35_–K_36_, because this position pair had a high chance of forming an OSC independent of codon bias in 85/100 sequences due to the presence of a NTN codon at position 35, followed by lysine AAN (Supplementary Figure S11).

**FIGURE 3:**
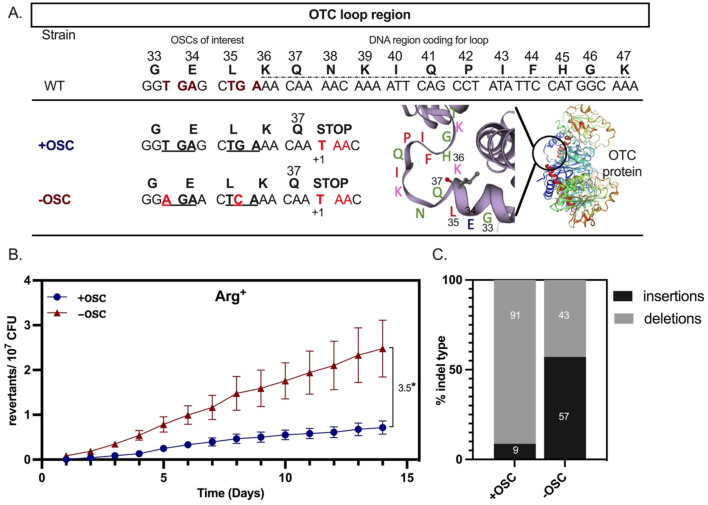
**(A)** Schematic of the *Bacillus subtilis* Otc structure and loop region K_36_–K_47_ taken from UniProt, the OSCs, and the changes made to remove them. **(B)** Results from the stationary phase assay comparing the +OSC and −OSC strains. Significance was determined using an unpaired t-test. Graphs show the mean of four replicates across the 14-day experiment, and error bars are SEM. **(C)** Sequencing results from independent Arg^+^ mutants that arose after day 5. We chose 23 for +OSC and 21 for −OSC independent Arg^+^ revertants for sequencing analysis, respectively. The Arg+ mutants were collected from the four SPM replicates. The % contribution of each indel type (insertions or deletions) was determined by diving the contribution of each by the total indel events for each strain.

Beyond *in silico* results, we established an experimental system to determine whether OSCs preceded the loop influence accumulation of spontaneous indel events and the production of functional Otc variants. Our studies focused on OSCs between the codons of amino acids 33–36 in the *B. subtilis* Otc (Figure 3A, top panel). In one case (−OSC), we introduced synonymous base substitutions in this DNA region to remove both OSCs while maintaining the amino acid sequence of Otc. In the second case (+OSC), we selected for indel events by inserting a +1T at codon 38 of the *argF* gene to generate an ochre stop (+1TAA), rendering the cells auxotrophic for arginine (Arg^−^) (Figure 3A). The +1 insertion was also introduced to the ORF in the −OSC strain; thus, both strains could be used in a Arg^−^→Arg^+^ selection system. Next, engineered *argF* variants were placed under an IPTG inducible promoter with low levels of basal transcription (Castillo-Hair et al., 2019). After IPTG induction, we measured the mutation frequency of indel events that restore arginine prototrophy during stationary growth (i.e., the generation of Arg^+^ colonies on minimal media lacking arginine) in the presence and absence of OSCs.

### Loss of OSCs increases Arg^+^ in stationary *B. subtilis* cells

We used a stationary-phase assay to determine how the presence or absence of OSCs directly upstream of the ArgF K_36_–K_47_ loop influences the accumulation of spontaneous indel events in the Otc loop during arginine starvation. In brief, we grew cells to stationary phase, plated them on minimal media lacking arginine, and scored Arg^+^ colonies over 14 days. We found that OSC removal increased the mutation frequency ∼3.5-fold (Figure 3B).

### Arg^+^ prototrophy arises by unique and common indel events, but their complexity is increased in the −OSC background

**FIGURE 4:**
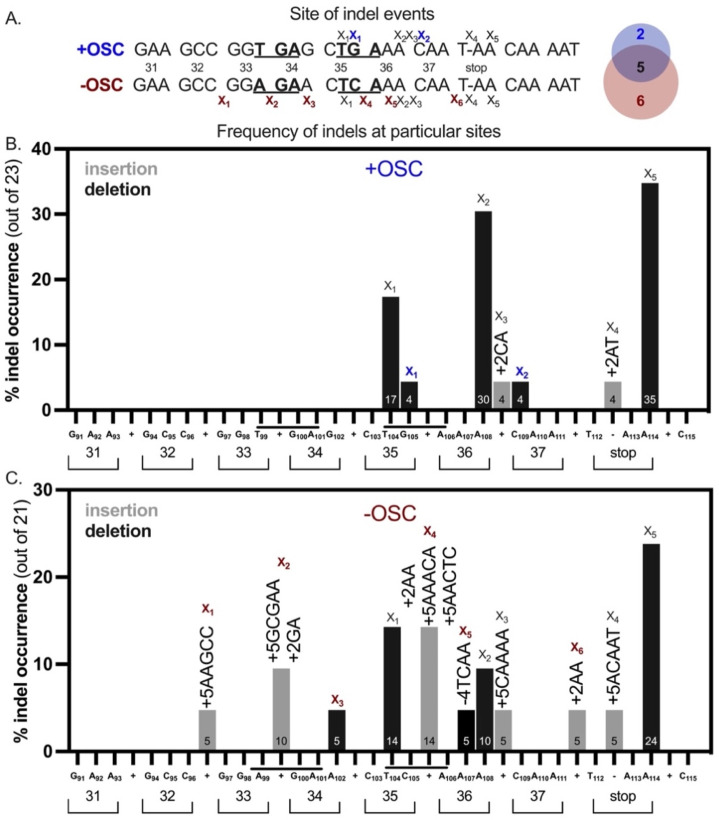
Loss of OSC increases unique indels in *Bacillus subtilis argF*protein loop region during stationary-phase. **(A)** Schematic of the sites (“x”) for indel events for each strain. An “x” directly below a base means that the base was deleted; insertion events were placed between codons. Venn diagram depicts the number of unique sites for each strain (+OSC: blue; −OSC: red) along with the common sites (black). **(B,C)** Bar graphs showing the % occurence of each indel event for each strain. The sequence and codon position is shown in the X-axis. The number inside the bar indicates the % occurrence of each bar. The gray bars indicate insertions, and the black bars indicate single-base deletions, with exception of one four-base deletion in the –OSC strain. The indel events shown above the bars indicate the indel event seen at each site.

**FIGURE 5:**
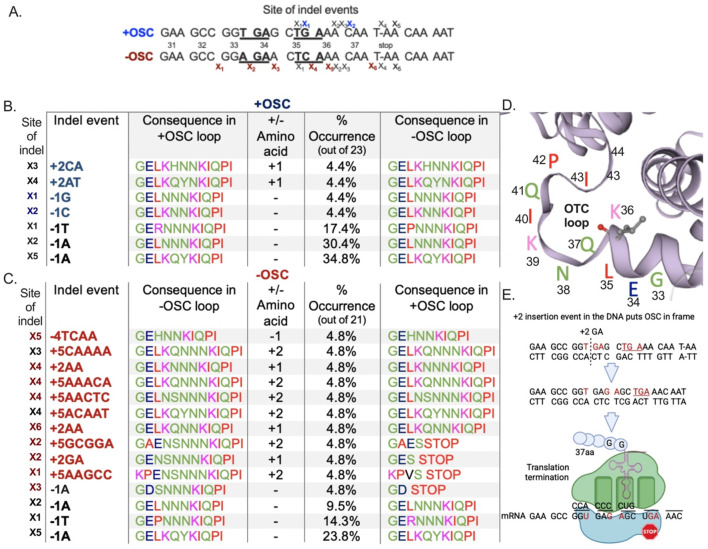
Impact of indel events on the amino acid sequence and length of the Otc protein loop region K_36_–K_47_. **(A)** Schematic of the sites (“x”) for indel events for each strain for reference. Table format showing results for the +OSC strain **(B)** and the −OSC strain **(C)**: indel site relative to the sequence in panel A (first column); indel event that occurred at the site (second column); consequences that the indel event had on the amino acid Otc loop (third and fourth columns); how common that indel event was among those sequenced (fifth column labeled % occurrence); consequence of an indel on the Otc loop in the opposite strain (sixth column). **(D)** Schematic of the amino acids found in the loop region of the *Bacillus subtilis*Otc protein. **(E)** Schematic recreating +2 GA (x_2_red) insertion observed in the −OSC strain in the context of the +OSC strain (OSCs in red font); the result is the indel even places an OSC in frame and terminates translation.

Because Arg^+^ prototrophy could result from deletion or insertion events, we sequenced the *argF* gene in stationary-phase Arg^+^ revertants and found that 57% of mutants in the −OSC strain had insertion events compared to 9% in the +OSC strain (Figure 3C). To better understand these differences in indel events, we determined the *site* and *type* of indel events in +/−OSC strains. Both strains had common indel sites (Figure 5AB, black x); two indel sites were unique to the +OSC strain (Figure 5A, blue x), and six were unique to the −OSC strain (Figure 5B, red x). Three unique sites in the −OSC strain occurred before codon 35 (Figure 5A). We also queried what indel events occurred in each strain and analyzed their frequency in the mutant population. Even when the indel site and type were the same (−1A) in both strains (Figure 4BC, black x_2_), the frequency varied (30% in +OSC and 10% in −OSC). Importantly, a single site contained several different indel events and was more common in the −OSC strain (Figure 5C red x_4_). The −OSC strain acquired more indel events downstream of codon 35, and the insertions were more complex (larger than two nucleotides) than those in the +OSC strain (Figure 5B).

### OSC loss promotes indel events that lengthen the Otc protein loop region

To better understand how OSCs affect genetic variation and its consequences in amino acid composition of the loop region, we examined the impact of indel events on the codon sequence in Arg^+^ mutants. We focused on how each indel event affected the codon composition and length of the Otc loop region relative to the WT protein. We identified a 12-amino-acid Otc loop region in *B. subtilis* spanning residues K_36_–K_47_ (Figure 5D) and calculated the percentage occurrence of specific indel event types in the sequenced mutant population (Figure 5B). Specifically, we identified: 1) the indel site (first column); 2) the indel event that occurred at the site (second column); 3) the consequences of the indel event on the amino acid Otc loop (third and fourth columns); 4) the frequency of that indel event among those sequenced (fifth column labeled % occurrence). Using *a sequence reconstruction* analysis, we also recreated the indel event in the opposite strain to examine its consequence on the Otc loop (sixth column). We found that the Otc loop region could tolerate deviations from WT loop length and composition, which is consistent with our amino acid sequence alignment showing that this region varies in loop size and composition across prokaryotes (Figure 1).

We found three indel events common to both strains that combined to represent the most frequent indel event in the strains (Figure 5). Of note, deletion events −1A (x_2_) and −1A (x_5_) occurred in the context of a di-adenine run (Figure 5A). Although the loop length of these indel events (−1T x_1_, −1A x_2_, −1A x_5_) did not deviate from the WT, they did change the amino acid composition. A −1 deletion event common to both strains (X_1_ −T in black) caused different amino acid composition in the loop (arginine 35R in +OSC strain, and proline 35P in the −OSC strain), both of which deviated from the WT sequence by three amino acids. Interestingly, this deletion comprised 17.4% of the total mutants in the +OSC strain and 14.3% in the −OSC strain.

By examining the codon sequence generated by indels unique to each strain, we found that the *B. subtilis* Otc loop region acquired codon sequences that deviated from the WT loop. Indel events that occurred uniquely in both strains resulted in shortening of the loop by one residue and lengthening of the loop by up to two amino acid residues. However, the −OSC strain accumulated larger indels than the +OSC strain at or downstream of codon 35, except for one four-base pair deletion; these genetic changes produced longer Otc loops (Figure 5). The unique indel events in both strains were detected at similar frequencies (4.4 or 4.8%) in the sampled mutant population, suggesting no differences in fitness costs between unique mutants.

To gain insight into why an indel event was found in one strain and not the other, we performed *a sequence reconstruction* analysis to recreate the indel event in the opposite strain (column six) and observed consequences on the loop. In this analysis, ∼36% (4/11) of unique indel events in the −OSC strain would not be possible in the +OSC strain because they would place an out-of-frame stop codon in frame, resulting in translation termination and a non-functional truncated Otc protein (Figure 5E).

### Mfd promotes indel mutations in the coding region for Otc loop K_36_–K_47_

**FIGURE 6:**
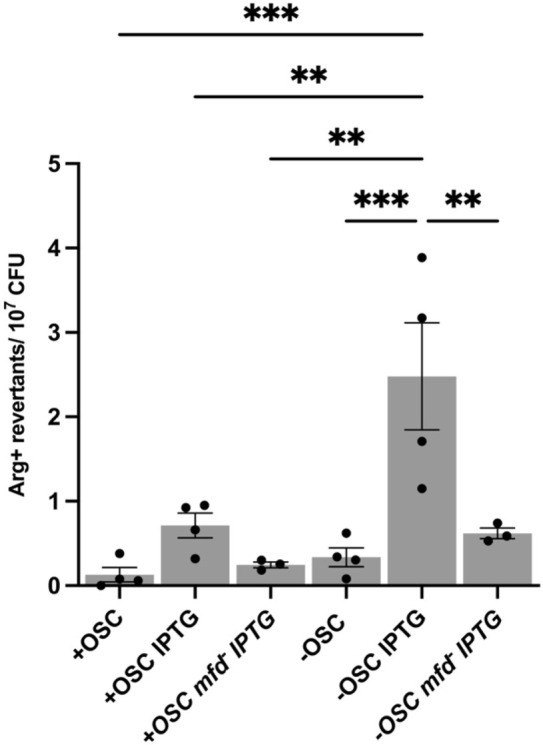
Results from the stationary-phase assay comparing the +OSC and −OSC strains +/− 0.1 mM IPTG and their *mfd* ^−^ counterpart. Bars are the mean of the total Arg^+^ mutants at the end of the experiment. Significance was determined using ordinary one-way ANOVA. Error bars are SEM.

We previously showed that Mfd and transcription levels are important drivers of base substitution mutations in the *B. subtilis* stationary phase (Martin et al., 2011). We also asked whether these factors affect indel production in our +/−OSC *argF* systems. In the absence of Mfd and IPTG, which induce transcription beyond basal promoter levels, Arg^+^ mutation frequency decreased in the −OSC strain (Figure 6). Since our previous studies showed that distinct mutagenic mechanisms occur in exponentially growing cells but not in stationary-phase cells (Leyva-Sánchez et al., 2020), we asked whether Mfd affected the ArgF^+^ mutation rate in growing *B. subtilis* strains with or without OSC. Consistent with stationary-phase cells, the −OSC strain had a higher mutation rate than the +OSC strain. Sequence analysis was also consistent with profiles seen in stationary-phase cells, with the −OSC strain having more complex insertion events in the loop K_36_–K_47_ (Supplementary Figures S8-S9). Importantly, 40% of the unique indel events observed in growing −OSC strain cells would not be possible in the +OSC strain because they would place an out-of-frame stop codon in frame, resulting in translation termination and a non-functional truncated Otc protein (Supplementary Figure S9). However, the effect of Mfd was opposite, with the results showing that the absence of Mfd increased the production of ArgF^+^ revertants in the +OSC strain. In contrast, no impact was observed in the −OSC strain. Thus, in growing *B. subtilis* cells, *mfd* prevents indel events at OSC, leading to *argF* ^−^ gene reversions.

## Discussion

In this study, we developed a genetic system that allowed us to determine the function of OSCs in bacterial genomes and whether they protect against frameshifts that alter the ORF and produce aberrant proteins, as speculated by some *in silico* studies. Unlike base substitutions, frameshifts resulting from insertion and deletion events disrupt the ORF and produce truncated or potentially toxic proteins. Additionally, studies have also found that genetic regions that acquire indels display an increased rate of base substitution mutagenesis, adding to their mutation load (Zhu et al., 2008; Mcdonald et al., 2011). Given the deleterious effects of indels, particularly in protein-coding genes (Coenye et al., 2006), we queried whether there are features in DNA associated with a decreased occurrence of indel events in coding sequences. We have provided evidence suggesting that codon sequences with the potential to generate premature stops (out-of-frame stop codons—OSCs) reduce the type and frequency of indel events at the expense of generating functional protein variants.

We first examined the codon sequence (argF gene) and protein structure of the Otc enzyme in *B. subtilis* and identified OSCs that fell in conserved amino acid codons in the protein. An analysis of 100 Otc protein sequences then showed that 14 OSCs in *B. subtilis* coincided with conserved residues from distantly divergent prokaryotes. The majority of OSCs in the *B. subtilis argF* ORF were TGA codons, followed by TAA. The TAG stop codon was rare, which is consistent with previous research showing that TGA and TGA-OSCs increased with GC content, while TAG and TAG-OSCs were inversely related (Wong et al., 2008; Korkmaz et al., 2014; Povolotskaya et al., 2012). Interestingly, the TAA stop codon is the most efficient translational stop and is often found at the end of highly expressed genes, like ribosomal genes, needed during rapid growth in many bacterial species, including *B. subtilis* (Korkmaz et al., 2014; Sharp and Bulmer, 1988). The appearance of TGA throughout *argF* may speak to the role of TGA during starvation conditions. Indeed, many of the genes involved in arginine biosynthesis, including *argF*, end with TGA or TAG instead of TAA. Additionally, it has been found that in the stationary phase, the ratio of RF2—the ribosomal termination factor that recognizes TGA—increases compared to its RF1 counterpart (Korkmaz et al., 2014; Eymann et al., 2002).

We identified a region of the Otc protein that encodes a flexible loop (residues 36–47 in *B. subtilis*) of variable length (11–21 amino acids) and amino acid composition across distinct bacterial phyla. This finding is consistent with studies in which protein loops are under relaxed selection pressure and tolerate more variation than regions that encode residues associated with substrate binding or found in the protein core (Blouin et al., 2004). Interestingly, this variable loop was flanked by conserved OSCs. Seligmann (2019) found that OSCs were enriched downstream of homopolymer regions, adding to evidence that OSCs may be enriched around DNA regions that are indel prone. Our results also showed that OSCs preceding the loop were less likely to be conserved in prokaryotes with the longest protein loop, consistent with OSCs being absent in metabolically diverse bacteria (Wong et al., 2008). Together, the data suggest that the presence of OSC negatively correlates with genetic variation.

To determine the effects of two OSCs (XTG_A and XXT_GA) that mapped immediately upstream of the Otc protein loop on indel frequency consequences on protein variation, we developed a genetic system that introduced a +1 frameshift stop at codon 38 (TAA) (rendering Otc non-functional and able to carry out arginine biosynthesis) and then created two derivative strains. The +OSC strain had the +1 frameshift and the OSCs remained intact, while the −OSC strain had the same frameshift but lacked the two OSCs. We then scored indel frequency events that restored Otc function and led to Arg^+^ on minimal media plates. We found that removing the OSCs upstream of the protein loop led to increased Arg^+^. By analyzing Arg^+^ mutants, we identified three common events that occur between codons 35 and 38 in strains that differ in the presence of OSC. Two of these events occurred at di-adenine sites that were common to both strains. However, indel events unique to each strain appeared in the codon 35–38 region. In the absence of OSCs, mutagenesis resulted in protein loop variants ranging from −1 to +2 amino acids in both growing- and stationary-phase cells. In contrast, these changes were not observed in the +OSC strain. We found that 36% of the unique indel events that occurred in the −OSC strain would not be tolerated in the +OSC strain because they would put the OSC in frame and terminate translation. Therefore, the OSC limited indel events that produced a functional ArgF protein. This was consistent in growing cells, where 40% of the −OSC unique indel events would not be tolerated in the +OSC sequence. It is also possible that differences in indel size and frequency between the OSC constructs at codons 35–38 could be due to their sequence context. Because *B. subtilis* does not have a strong codon bias (Ogasawara, 1985) and our sequence change did not introduce polymer runs, no sequence differences in this region would account for increased indel frequency in −OSC. However, sequence changes to remove the OSC may influence the mechanism of indel production in a stationary phase.

Mechanistically, indel events are thought to arise from DNA polymerase slippage on repetitive sequences (Levinson and Gutman, 1987) and nonhomologous end-joining (NHEJ) repair of double-stranded breaks (Gong et al., 2005; Lieber et al., 2003). In bacterial studies, regions with long mononucleotide sequence repeats were excluded from coding regions (Coenye et al., 2006), suggesting that indel occurrence was limited to non-coding regions. Interestingly, in stationary-phase cells, we showed that Mfd promotes indel events, resulting in gain-of-function genetic events in the −OSC strain that influence indel frequencies in coding regions. Mfd is characterized as a protein that rescues RNAP when it is stalled on the template strand due to encountering a lesion, although this definition has been evolving (Deaconescu, 2021). In our laboratory, we have found that Mfd promotes base substitutions at transcribed regions in stationary-phase cells, and mutagenesis correlates with transcription level (Ross et al., 2006; Martin et al., 2021). However, in growing +Otc cells, Mfd prevented the production of ArgF^+^ reversions. In actively replicating cells, the mismatch repair system may prevent mutagenic events promoted by Mfd during stationary-phase growth when *mutSL* expression is limited (Pedraza-Reyes and Yasbin, 2004.

The Mfd repair factor preferentially recruits repair machinery to transcribed regions, working together with error-prone DNA polymerases to promote mutations and indel events that produce loss-of-function phenotypes (Villegas-Negrete et al., 2017). Mfd was also shown to promote mutations in genes encoded in the lagging strand during DNA replication in growing cells and is recruited to hard-to-transcribe regions (Merrikh et al., 2016; Ragheb et al., 2021). Based on these observations, we hypothesize that during the stationary phase, Mfd recruits PolIV/DinB to promote gain-of-function indel events in genes with increased transcription. Studies have shown that DinB has a sequence preference, which could explain some differences between +OSC and −OSC strains.

While some studies have identified the correlated occurrence of indel events and translation bypass, the dependence on Mfd, which has been implicated in mutagenesis including indel events, suggests that translation bypass is not likely to play a role in our study. Additionally, the sequences used here do not contain homopolymer runs prone to bypassing, and the Arg+ mutants all contain indels that restore the frame to produce a functional Otc (Rockah-Shmuel et al., 2013; Villegas-Negrete et al., 2017).

Our mutagenic assays showed that OSCs limit the formation of spontaneous indel events and generate flexible loop variants in the *B. subtilis* Otc enzyme, both by terminating events that place the OSC in frame and by OSC independent methods that may influence underlying mutagenic mechanisms. Thus, our results highlight an evolutionary trade-off between preventing the translation of aberrant gene products and forming new protein domains by extending flexible loop domains, particularly in highly transcribed genes subject to transcription-coupled repair. Our results are consistent with a model in which surface loops, which are less constrained by selection (rapid protein evolution) than regions that encode active site residues, “sample” indel events that alter the number of amino acids in a protein in a “near-neutral selection fashion”, and eventually grow into new protein domains (Blouin et al., 2004). Our study provides some of the first experimental data that OSCs have a function in bacterial genomes and that they may function as genetic constraints on protein evolution.

## Data Availability

The raw data supporting the conclusions of this article will be made available by the authors without undue reservation.
