# Supplementary material for: Genetic constraints on protein loop evolution: out-of-frame stop codons limit Bacillus subtilis stationary-phase mutagenesis
Source: Front Mol Biosci. 2026 Mar 9;13:1714028. doi: 10.3389/fmolb.2026.1714028 (PMC13006647; doi:10.3389/fmolb.2026.1714028)
Supplement: Supplementary file 1 [file DataSheet1.docx]

Supplemental

| 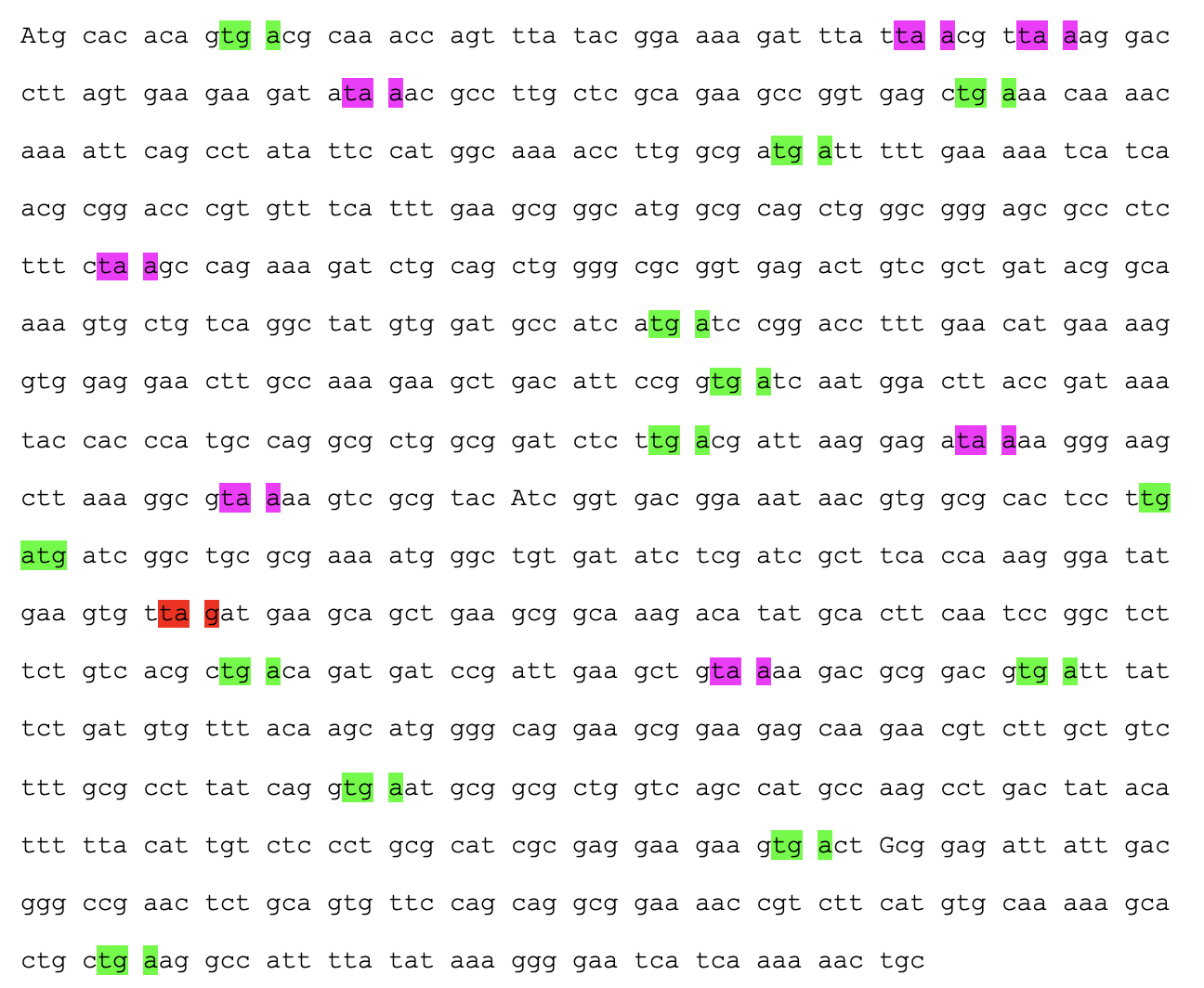 | | |
| --- | --- | --- |
|  | Pattern search | Count |
| Ochre stop TAA (purple) | XTA AXX | 7 |
| Opal stop TGA (green) | XTG AXX | 13 |
| Amber stop TAG (red) | XTA GXX | 1 |
|  | Total | 21 |

**Figure S1**: Summary of OSC analysis deleting 1 nucleotide. The sequence is the complete *argF* ORF with the OSCs highlighted in different colors depending on the type of stop. Ochre (TAA) stops are highlighted purple, Opal (TGA) stops are highlighted green, and Amber (TAG) stops are highlighted red. In this analysis the OSC could be placed in frame by a -1 deletion event at the beginning of the codon or a +2 insertion before the stop.

| 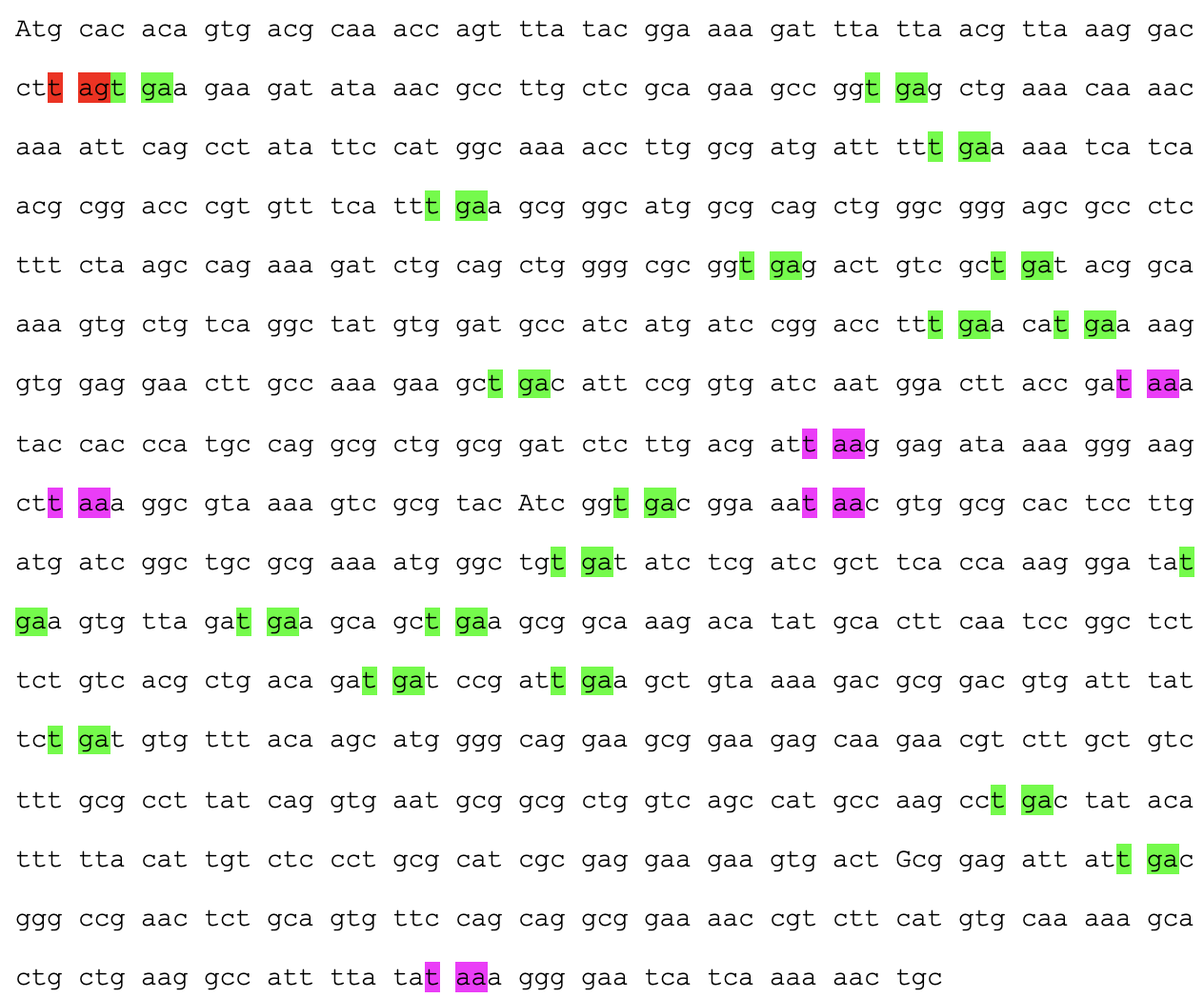 | | |
| --- | --- | --- |
|  | Pattern search | Count |
| Ochre stop TAA (purple) | XXT AAX | 5 |
| Opal stop TGA (green) | XXT GAX | 19 |
| Amber stop TAG (red) | XXT AGX | 1 |
|  | Total | 25 |

**Figure S2**: Summary of OSC analysis inserting 1 nucleotide. The sequence is the complete *argF* ORF with the OSCs highlighted in different colors depending on the type of stop. Ochre (TAA) stops are highlighted purple, Opal (TGA) stops are highlighted green, and Amber (TAG) stops are highlighted red. In this analysis the OSC could be placed in frame by a +1 insertion event before the stop or a -2 deletion event.

**
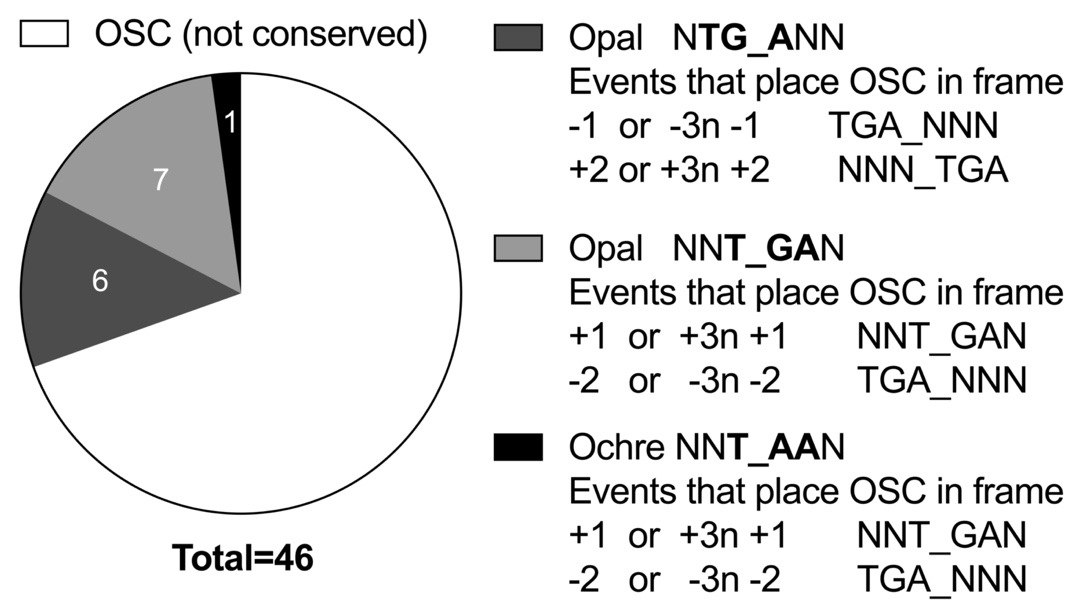
**

**Figure S3:** Pie chart showing the distribution of conserved and non-conserved OSCs found in *argF* combining the -1 and +1 analysis. Conservation was based on a sequence alignment of 100 Otc proteins; if >50 sequences had the same amino acid that position was conserved. The 14 conserved OSCs are further broken down by type of stop codon and the indel event needed to place the OSC in frame. In six of the fourteen opal stops, a -1 deletion results in a translational stop (NTG_AXX 🡪 delete N: TGA_XXX). In addition to the -1 deletion, a +2 insertion would also place the OSC in frame (XTG_AXX 🡪 insert NN: XNN_TGA). In the remaining 7 opal stops, a +1 insertion event results in a translational stop (XXT_GAX 🡪 insert N: XXN_TGA). In addition to the +1 insertion, a -2 deletion would also place the OSC in frame (NNT_GAX 🡪 delete NN: TGA_XXX). For the ochre stop, a +1 insertion place the OSC in frame (XXT_AAX 🡪 insert N: XXN_TAA), a -2 deletion would also place the ochre stop in frame (NNT_AAX 🡪 delete NN: TAA_XXX). Amber stops (TAG) only comprised 2/46 of the OSCs found in the *argF* ORF, and the amino acid codons encompassing these OSCs were not conserved.

***B. subtilis* OTC Structure analysis.**

Structure analysis began by searching the UniProtKB (Consortium 2015) database for *B. subtilis* ornithine carbamoyltransferase (Otc) entries. We found an entry with a yellow icon indicating it belonged to the Swiss-Prot section of UniProt and had been reviewed. At the time of the search, the entry was last modified in 2018 (P18186 (OTC_BACSU). Important binding sites for the *B. subtilis* Otc were annotated in UniProt and noted to be inferred from the following UniProt entries which all included x-ray protein structures: [P04391 (OTC1_ECOLI)](https://www.uniprot.org/uniprot/P04391),  [P9WIT9 (OTC_MYCTU)](https://www.uniprot.org/uniprot/P9WIT9), and [Q8DCF5 (OTC_VIBVU)](https://www.uniprot.org/uniprot/Q8DCF5). To determine the Otc loop regions, we referenced the Otc *B. subtilis* Swiss-Model-homology model linked to the UniProt entry; this model was built using a *B. anthracis* OTC x-ray structure as a reference Q81M99. Additionally, we used the same UniProt entries that were used to determine binding sites since they all contained a linked x-ray protein structure. We also referenced Sankaranarayanan et al 2008 (Sankaranarayanan et al. 2008) which was the reference for the Mycobacterium entry (OTC_MYCTU). This publication contained an Otc alignment comparing the *Mycobacterium* Otc sequence and secondary structure to 5 other sequences with published structures including the human Otc. From this structure analysis, we established that there was a loop region in *B. subtilis* Otc that spanned amino acids 36-47. We also collected the UniProT Otc sequences for all the structures referenced in the structure analysis and used Clustal Omega multiple sequence alignment program to align the protein sequences. The results showed that the protein loop region was not conserved in length or amino acid sequence.


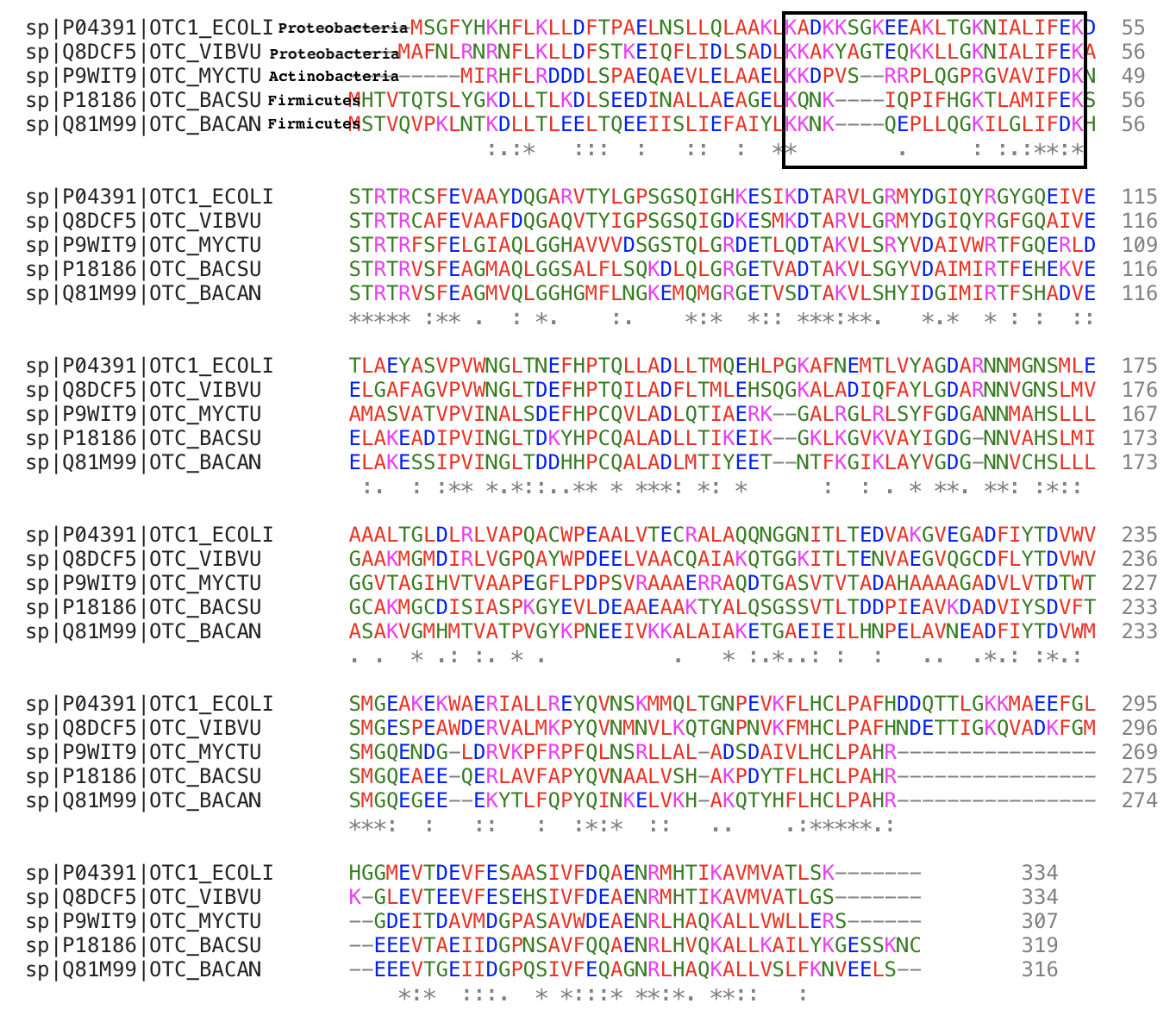


**Figure S4.** Clustal Omega multiple sequence alignment depicting the Otc protein sequences of several distantly related bacteria with publicly available OTC structures we used in this study to identify protein features. The region boxed in black highlights the OTC loop region of interest.


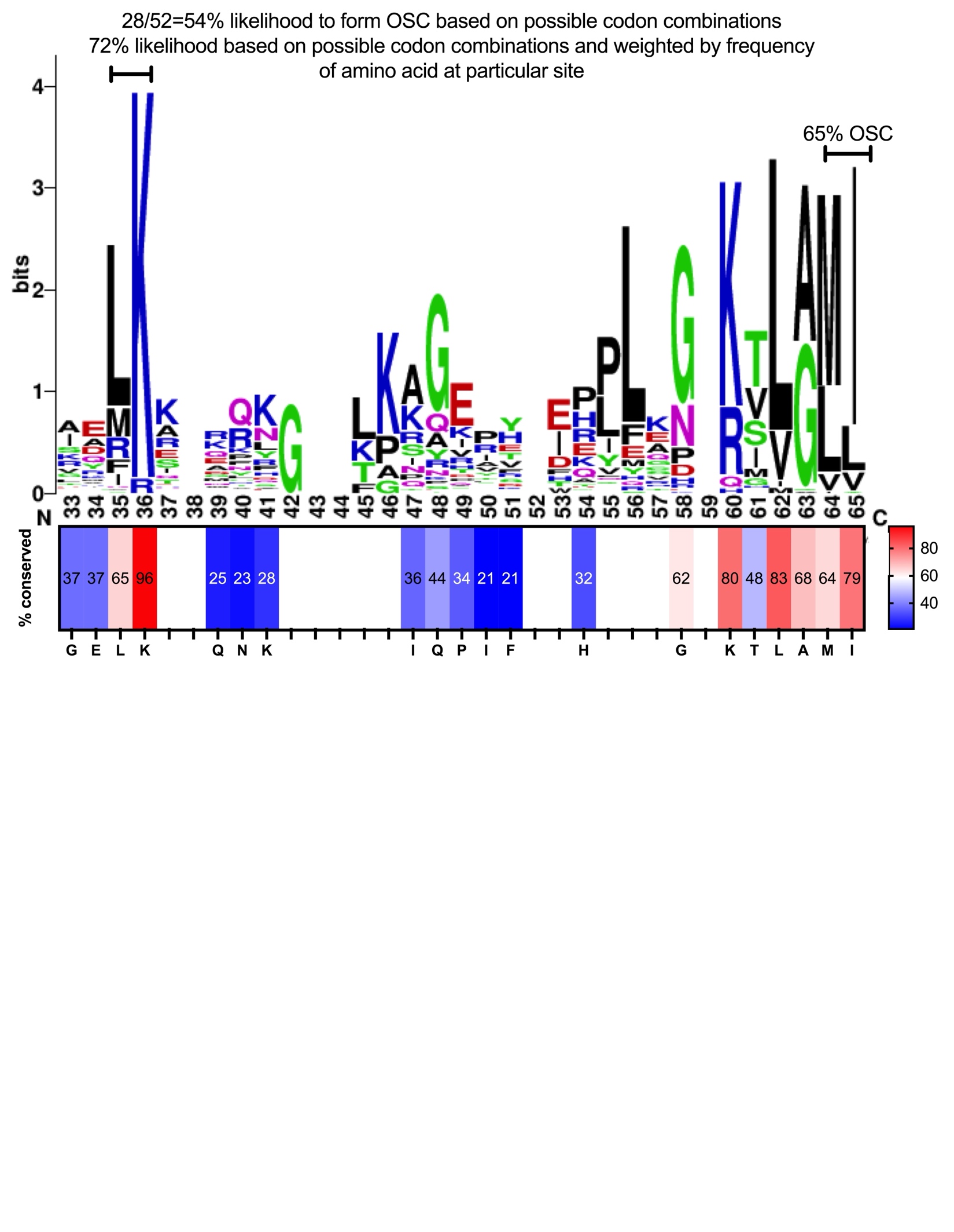


**Figure S5:** WebLogo results showing all amino acid residues present in the loop region and their frequency in the 100 sequences (the size of the letters on the Y axis indicates frequency). We define the loop spanning position 36K-60K for this analysis. The X axis represents the position in the alignment and how conserved that position is. The heat map on the X-axis indicates how many sequences out of the 100 had the same residue as the *B. subtilis* residue indicated by the letter under the heat map. The % value above positions 36-37 and 64-65 indicates the likelihood that those positions will contain an OSC based on the amino acids seen at that position and their codons. If the % value indicates it was weighted, this means we considered the frequency of the amino acid at that position along with the codon possibilities.

For example, L was found in 65 of 100 sequences (frequency .65) and K was found in 96 of 100 sequences (frequency .96). We then calculated the frequency of each codon for each amino acid (L = 6 codon possibilities 100/6 codons=16.6 = .16 frequency for each codon). For K (K= 2 codon possibilities 100/2=50 =.5 frequency for each codon). Example calculation for one codon: (.65L x .16 CTG) x (.96K x .5 AAA)= 5.20% CTG_AAA.

This analysis considered all possible combinations of amino acid pairs for the given position.

In a second analysis we calculated the likelihood of each of the 100 species to form an OSC at position 35, 36 and then calculated the likelihood of the 100 sequences. Like our 1^st^ analysis we found that there was a 74% chance of forming an OSC. This analysis excluded non-occurring amino acid pairs.

**
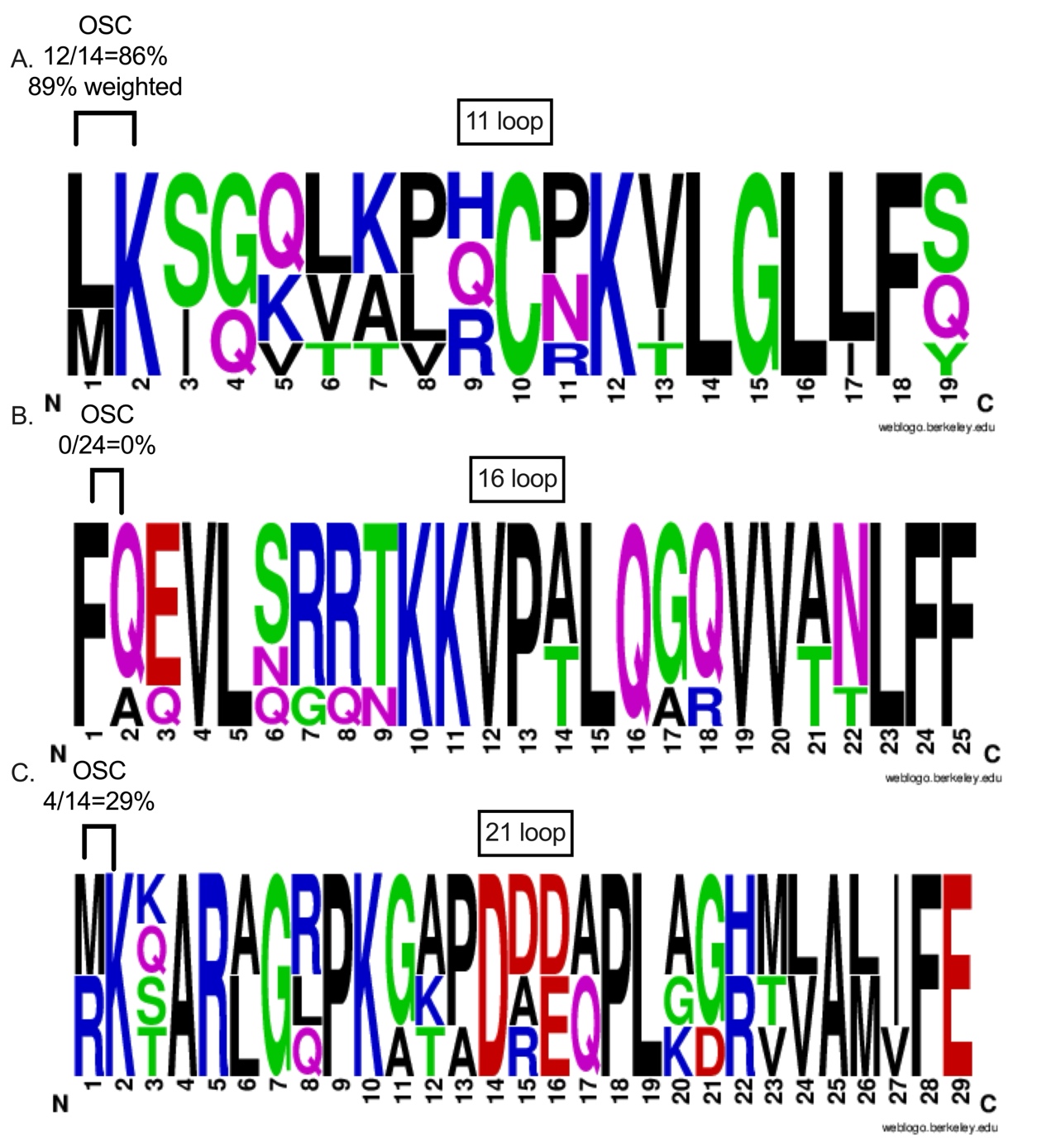
**

**Figure S6:** WebLogo results showing all amino acid residues present in the loop region and their frequency among other amino acids at that position. Panel A shows the WebLogo results for six sequences that had a loop size of 11. Panel B shows the WebLogo results for 5 sequences that had a loop size of 16 excluding *B. subtilis*. Panel B shows the WebLogo results for 4 sequences that had a loop size of 21. The % value indicates the likelihood that those positions will contain an OSC based on the amino acids seen at that position and their codon. If the % valued indicates it was weighted, this means we considered the frequency of the amino acid at that position along with the codon possibilities.

**
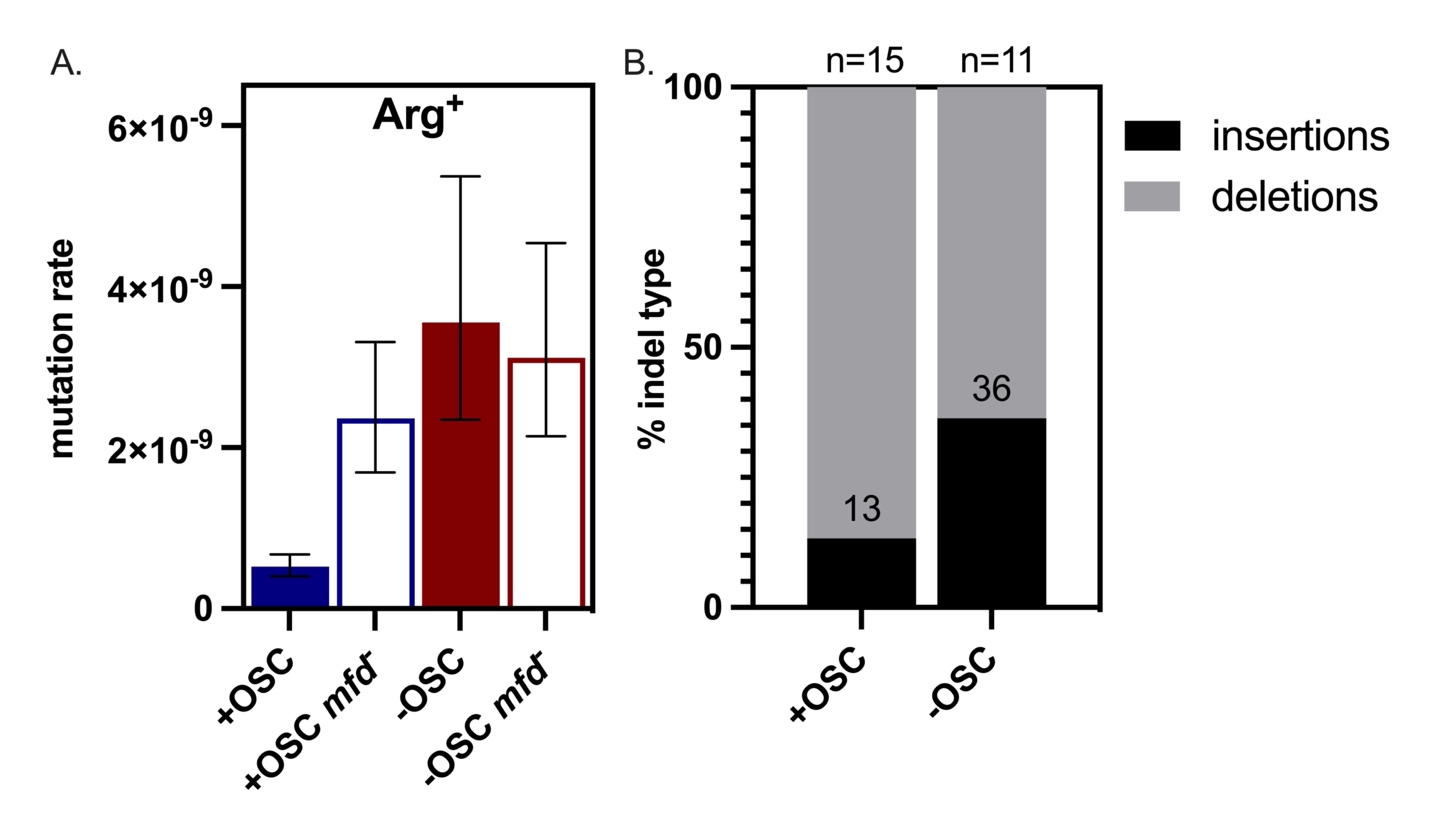
**

**Figure S7:** Results from the fluctuation test comparing mutation rates between the +OSC and the -OSC strains. Mutation rate was determined using the Lea-Coulson method of the median and error bars are CI (95). B. Sequencing results for the Arg^+^ that arose during growth, colonies picked were from individual plates to avoid picking clones.

**
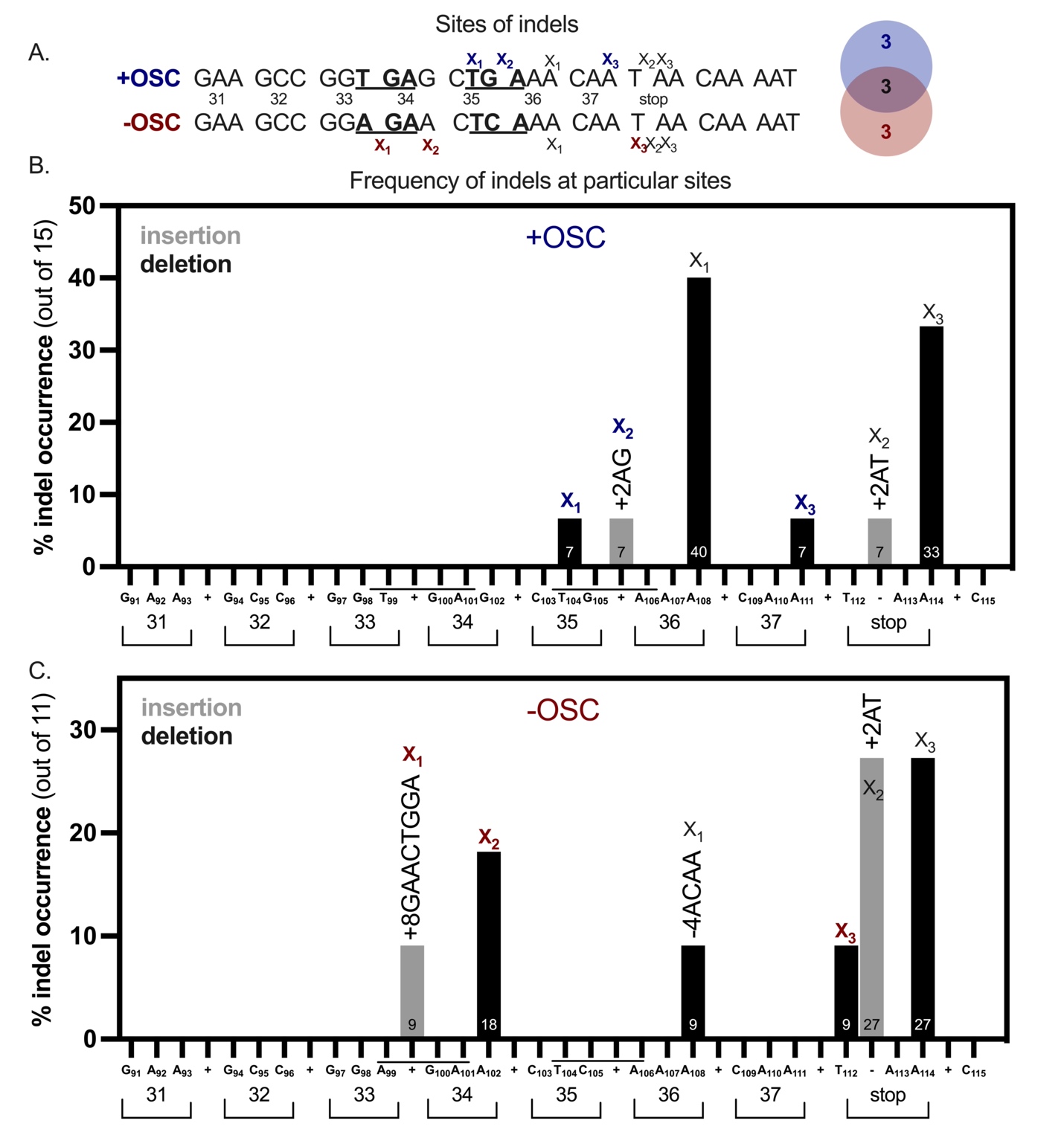
**

**Figure S8:** Loss of OSC increases unique indels in *B. subtilis* growing cells. A Schematic depicting the sites (x) for indel events for each strain. The Venn diagram depicts the number of unique sites for each strain (+OSC-blue, -OSC-red) along with the common sites (black). B. Bar graph showing the % occurrence of each indel event. The sequence and codon position is shown in the x-axis. The number inside the bar indicates the % occurrence of each bar. The gray bars indicate insertions, and the black bars indicate single-base deletions. The indel events shown above the bars indicate the indel event seen at each site.


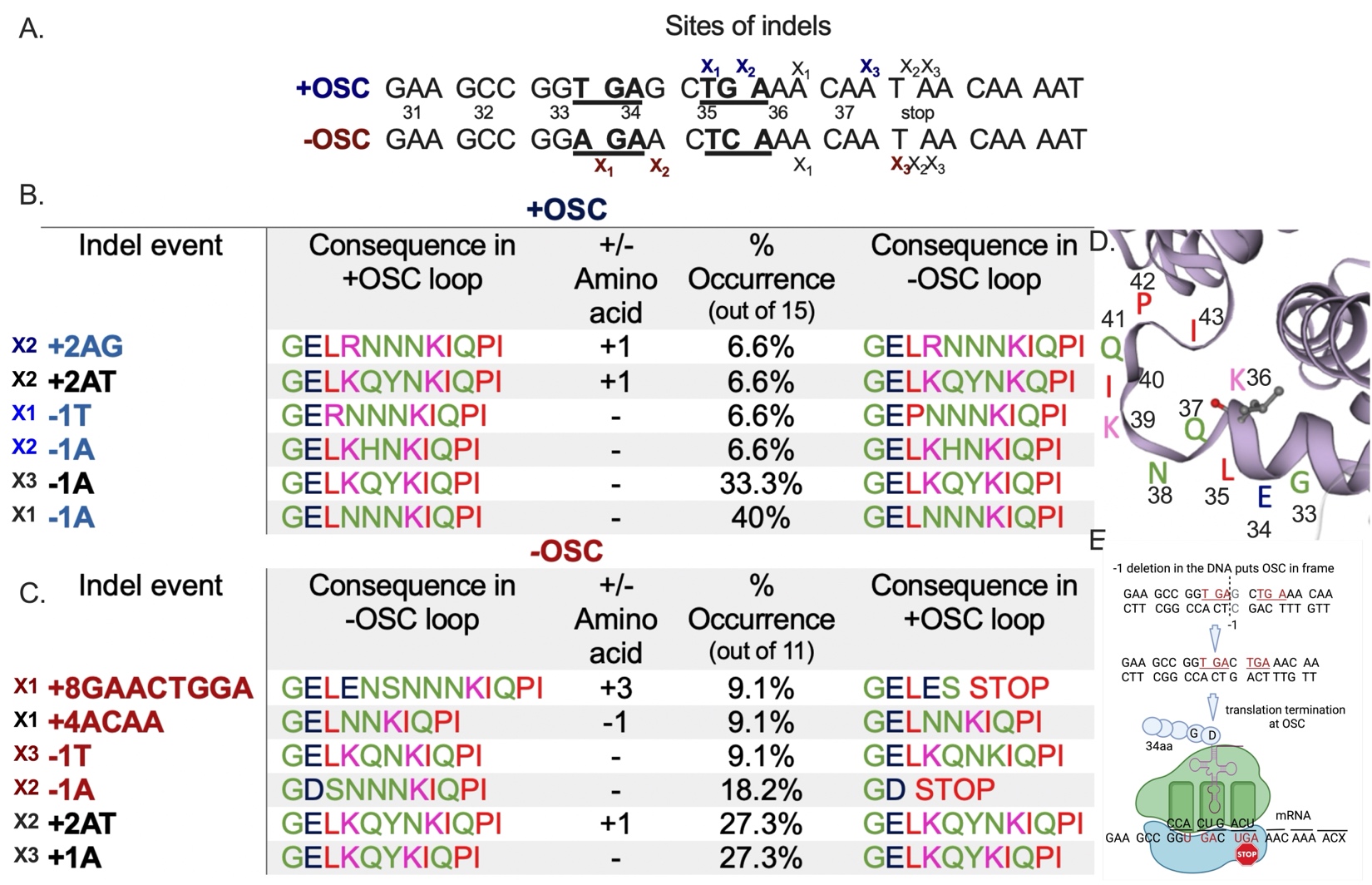


**Figure S9**: The consequence of indel events on the Otc protein loop region. A Schematic depicting the sites (x) for indel events for each strain for reference. Table format: indel site relative to the sequence found in panel A (first column), the indel event that occurred at the site (second column), the consequences that indel event had on the amino acid Otc loop (third and fourth column) and how common that indel event was among those sequenced (fifth column labeled % occurrence). Consequence of an indel on the Otc loop in the opposite strain (sixth column). D. Schematic depicting the amino acids found in the loop region of the *B. subtilis* Otc protein. E. Schematic that recreates -1 (x_2_ red) insertion observed in the -OSC strain in the context of the +OSC strain (OSCs in red font), the result is the indel even places an OSC in frame and terminates translation.


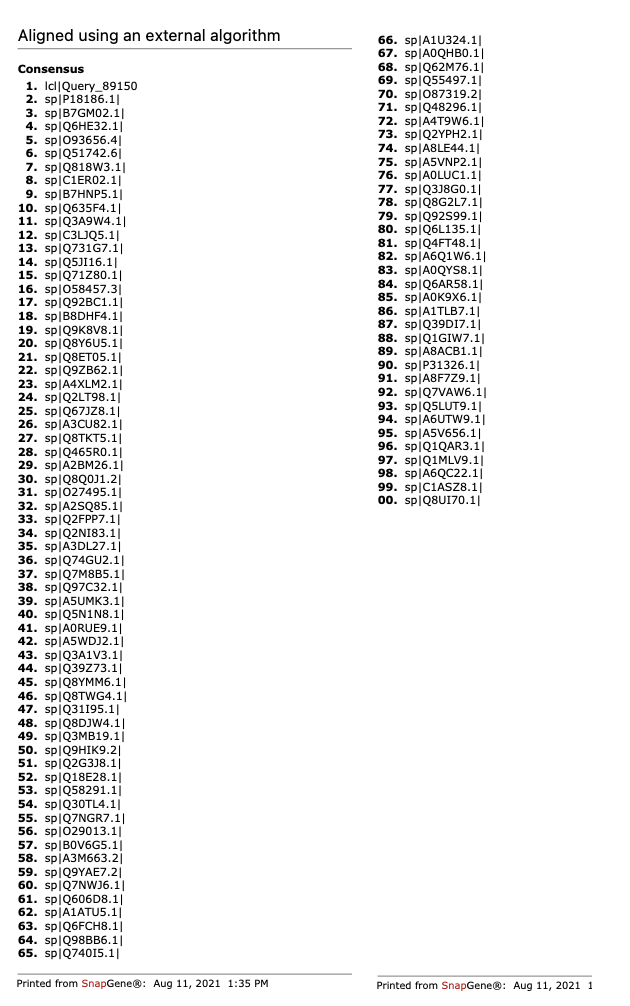


Figure S10: Identification of 100 sequences used in this study.


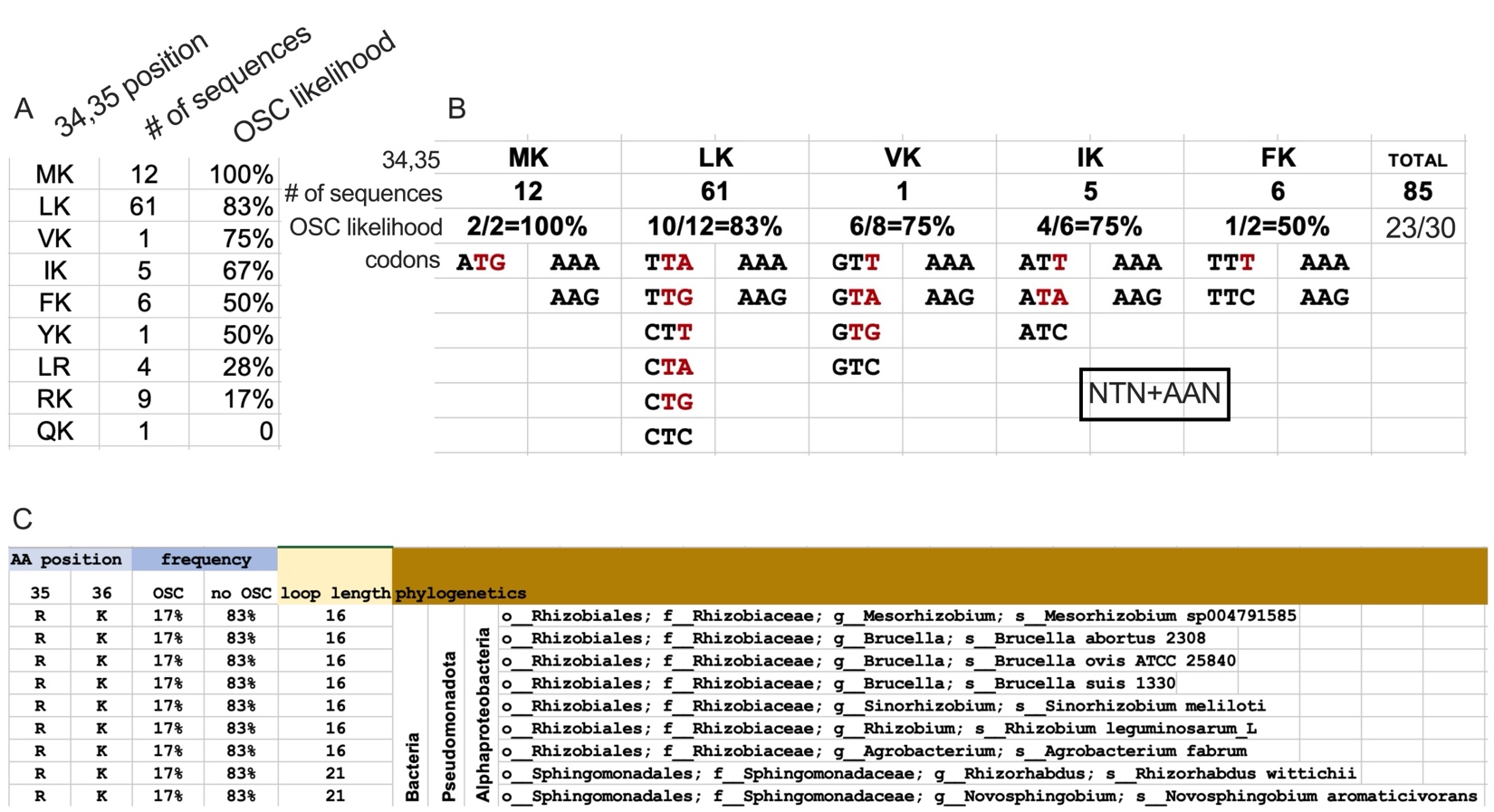


**Figure S11:**

To generate table A, we calculated the likelihood of each of the 100 species to form an OSC at position 35, 36 and then grouped them based on amino acid pairs. B. 85 of the 100 sequences have a high chance of forming an OSC because they have NTN at position 35 followed by lysine AAN. C. The amino acid pair RK was not likely to form an OSC. Those that had RK belonged to a similar phylogenetic group and had a similar larger loop length.

**Consortium, The Uniprot. (2015) ‘UniProt: A hub for protein information’, Nucleic Acids Research, 43, pp. 204–212.** [**https://doi.org/10.1093/nar/gku989**](https://doi.org/10.1093/nar/gku989)**.**

**Sankaranarayanan, R., Cherney, M.M., Cherney, L.T., Garen, C.R., Moradian, F. and James, M.N.G. (2008) ‘The crystal structures of ornithine carbamoyltransferase from Mycobacterium tuberculosis and its ternary complex with carbamoyl phosphate and L-norvaline reveal the enzyme’s catalytic mechanism’, Journal of Molecular Biology, 376(4), pp. 1052–1063.** [**https://doi.org/10.1016/j.jmb.2007.11.025**](https://doi.org/10.1016/j.jmb.2007.11.025)**.**
